# Examining the association of social risk with heart failure readmission in the Veterans Health Administration

**DOI:** 10.1186/s12913-021-06888-1

**Published:** 2021-08-26

**Authors:** Charlie M. Wray, Marzieh Vali, Louise C. Walter, Lee Christensen, Wendy Chapman, Peter C. Austin, Amy L. Byers, Salomeh Keyhani

**Affiliations:** 1 Department of Medicine, University of California, San Francisco, USA; 2 Division of Hospital Medicine, San Francisco Veterans Affairs Medical Center, University of California San Francisco, 4150 Clement Street, San Francisco, CA 94121 USA; 3 Northern California Institute for Research and Education, San Francisco Veterans Affairs Medical Center, San Francisco, USA; 4 Division of Geriatrics, San Francisco Veterans Affairs Medical Center, San Francisco, USA; 5 Department of Biomedical Informatics, University of Utah, Salt Lake City, USA; 6 Centre for Digital Transformation of Health, University of Melbourne, Melbourne, Victoria Australia; 7 Institute of Health Policy, Management and Evaluation, University of Toronto, Toronto, Canada; 8 Department of Psychiatry, University of California, San Francisco, USA; 9 Mental Health Services, San Francisco Veterans Affairs Medical Center, San Francisco, USA; 10 Division of General Internal Medicine, San Francisco Veterans Affairs Medical Center, San Francisco, USA

**Keywords:** Social Risk Factors, Heart Failure, Readmissions, Veterans Affairs

## Abstract

**Background:**

Previous research has found that social risk factors are associated with an increased risk of 30-day readmission. We aimed to assess the association of 5 social risk factors (living alone, lack of social support, marginal housing, substance abuse, and low income) with 30-day Heart Failure (HF) hospital readmissions within the Veterans Health Affairs (VA) and the impact of their inclusion on hospital readmission model performance.

**Methods:**

We performed a retrospective cohort study using chart review and VA and Centers for Medicare and Medicaid Services (CMS) administrative data from a random sample of 1,500 elderly (≥ 65 years) Veterans hospitalized for HF in 2012. Using logistic regression, we examined whether any of the social risk factors were associated with 30-day readmission after adjusting for age alone and clinical variables used by CMS in its 30-day risk stratified readmission model. The impact of these five social risk factors on readmission model performance was assessed by comparing c-statistics, likelihood ratio tests, and the Hosmer-Lemeshow goodness-of-fit statistic.

**Results:**

The prevalence varied among the 5 risk factors; low income (47 % vs. 47 %), lives alone (18 % vs. 19 %), substance abuse (14 % vs. 16 %), lacks social support (2 % vs. <1 %), and marginal housing (< 1 % vs. 3 %) among readmitted and non-readmitted patients, respectively. Controlling for clinical factors contained in CMS readmission models, a lack of social support was found to be associated with an increased risk of 30-day readmission (OR 4.8, 95 %CI 1.35–17.88), while marginal housing was noted to decrease readmission risk (OR 0.21, 95 %CI 0.03–0.87). Living alone (OR: 0.9, 95 %CI 0.64–1.26), substance abuse (OR 0.91, 95 %CI 0.67–1.22), and having low income (OR 1.01, 95 %CI 0.77–1.31) had no association with HF readmissions. Adding the five social risk factors to a CMS-based model (age and comorbid conditions; c-statistic 0.62) did not improve model performance (c-statistic: 0.62).

**Conclusions:**

While a lack of social support was associated with 30-day readmission in the VA, its prevalence was low. Moreover, the inclusion of some social risk factors did not improve readmission model performance. In an integrated healthcare system like the VA, social risk factors may have a limited effect on 30-day readmission outcomes.

**Supplementary Information:**

The online version contains supplementary material available at 10.1186/s12913-021-06888-1.

## Background

Heart failure (HF) is a leading cause of hospitalization in the Veterans Health Administration (VA) with more than 17,000 Veterans hospitalized every year [1]. Because readmissions for HF are so common and costly in older populations, the VA closely tracks 30-day readmission rates. Understanding which clinical and social factors are predictive of readmission is important in reducing readmissions and improving health and health outcomes.

In non-VA populations, previous studies have found that social factors such as use of Medicaid insurance, low income, being unmarried, and high-risk behaviors (e.g., smoking and substance abuse) are associated with hospital readmission [2–6]. Yet, a recent systematic review found that among 52 articles that examined the effect of social factors on risk of readmission [3], only one examined their impact within the VA health care system [7]. Moreover, in this study the authors only assessed one social risk factor (rurality), leaving a gap in our understanding of what social risk factors impact readmission risk within the VA.

As the nation’s largest integrated health care system, the VA provides robust clinical and social support services that include: Patient-Aligned Care Teams (PACT) for all Veterans and specialty-focused PACTs for Homeless Veterans, extensive mental health support services, housing assistance, and transport assistance among many other services. Understanding the impact of social risk on heart failure readmissions in a health care system that attempts to guard against the adverse effects of several social determinants of health is unknown. The goal of this study was to describe the association of five social risk factors (living alone, lack of social support, marginal housing, substance abuse, and low income) thought to be important in older populations, and not readily available in administrative databases on HF readmissions in a VA cohort. Such work could be helpful for the VA in understanding which non-clinical factors are impacting HF readmissions.

## Methods

### Data source and sample

Using VA administrative and Medicare claims data acquired from the VA Corporate Data Warehouse, we constructed a national cohort of Veterans among 10,761 Veterans, aged 65 and older, who were hospitalized for HF in 2012. Patients’ respective inpatient Medicare claims data were obtained from the Medicare Provider Analysis and Review (MedPAR) and Outpatient files. All Medicare records were linked to VA records via scrambled Social Security numbers and were provided by the VA Information Resource Center (VIReC). Patients were identified by a primary discharge diagnosis from VA administrative data by using International Classification of Disease, Ninth Revision, Clinical Modification (ICD-9-CM) codes. Because three of the assessed social risk factors (lives alone, lacks social support, and marginal housing) were not widely captured in administrative data during our assessment period, we randomly selected 1,500 Veterans from this cohort to allow for chart abstraction of these variables.

### Dependent variable

We followed model specifications outlined by the Center for Medicare and Medicaid Services (CMS) as the VA currently uses a CMS-based model to track hospital readmissions and for hospital profiling. Briefly, this model was approved by the National Quality Forum, to estimate hospital-specific readmission rates for Medicare patients hospitalized with HF. The model was developed and validated with Medicare administrative claims data and determined whether estimates from the claims model were good surrogates for the results of a medical record model. Like the CMS model, our main dependent variable was unplanned, all cause, 30-day readmission rates. All readmissions were defined as a subsequent inpatient admission to any acute care facility in either a VA or non-VA facility within 30-days of discharge from the index HF hospitalization, as outlined in the CMS technical documents used to define HF readmission [8].

### Predictor variables

#### Demographic and clinical factors

We identified patient age and the 32 clinical variables that are utilized in the CMS HF readmission model for all-cause HF readmissions [8]. All variables were identified in administrative data 1 year prior to the index admission. Sex was not included as few female Veterans were admitted for HF. Race was not included as the CMS model does not include race and we did not want to alter the underlying base model with which we would be comparing.

#### Social risk factors

Because administrative data lacks information on several common social risk variables, we utilized two methods to extract patients’ social risk: (1) manual chart abstraction, and (2) administrative coding (e.g., ICD-9-CM). Using chart review, we extracted data on three variables (social support, housing, and living situation [i.e., living alone]) with limited or no data in administrative datasets. To do so, we created a manual to help chart abstractors identify measures of social risk from the medical record (See Supplement for more detail). Each variable was extracted by a pair of reviewers, with a third reviewer adjudicating any disagreements. Briefly, presence or lack of social support was defined based on the following criteria: (a) specific mentions of social support (e.g., someone brings the patient to appointments), (b) specific mentions of lack of social support (e.g., no social support, social isolation), or (c) indirect evidence of lack of social support (e.g., patient lacks a ride post colonoscopy). For the living alone variable, we identified whether there was direct evidence (e.g., patient lives alone) or indirect evidence that the patient did not live alone (e.g., lives with a family member). For the housing variable, we considered a patient marginally housed if they lacked a fixed, regular, and adequate nighttime residence (e.g., sleeping on a friend’s couch) or were noted to be homeless. Those who lacked such characteristics were categorized as adequately housed. The presence of each variable was assessed in the year prior to admission.

We used ICD-9-CM diagnosis codes to assess for substance abuse (e.g., drug and alcohol abuse), using previously described methods [9]. To assess for low income status, we classified someone as low income if their VA co-payment was waived on the basis of their means test evaluation [10].

### Statistical analysis

The means (continuous variables) and prevalence (categorical variables) of patient characteristic were calculated separately for readmitted and non-readmitted patients. Using two multivariable logistic regression models, we examined whether any of the social risk factors were associated with 30-day readmission after adjusting for age alone (Model 1) and clinical variables used by CMS in its 30-day risk stratified readmission model (Model 2). Model 1 was examined to assess if social risk factors alone could be as predictive of readmission as commonly assessed clinical factors (found in Model 2).

We also calculated: (a) how the inclusion of these social risk factors would impact each models’ performance by calculating a c-statistic, (b) the effect of the addition of social risk factors on model fit through a likelihood ratio test, and (c) whether the observed event rate matches the expected event rate in each model (i.e. calibration) through the use of the Hosmer-Lemeshow goodness-of-fit (HL GOF) statistic.

## Results

Of the 1,500 chart-reviewed patients, 312 (21 %) were readmitted within 30 days of hospital discharge. Of these, most were white (75 %), had a history of coronary artery bypass procedure (79 %), heart failure (96 %), cardiac arrhythmia (77 %), coronary artery disease (78 %), and renal failure (70 %). Rates of these conditions were not substantially different from those who were not readmitted and the full sample (10,761) from which this cohort were pulled from. Readmission rates was also similar between the full sample (20 %) and the chart-reviewed cohort (21 %). Among administratively extracted variables, the prevalence of the assessed social risk factors were similar between groups, with almost half (47 %) having low income, and many suffering from substance abuse (14 and 16 %) among readmitted and non-readmitted patients, respectively.

Among variables extracted through chart review, there was little difference in the prevalence of living alone (18 and 19 %) between groups. There were differences in the prevalence of lacks social support (2 and 0.5 %) and in those who were marginally housed (0.6 and 2 %) among readmitted and non-readmitted patients, respectively (Table 1).

**Table 1:** Characteristics for Patients Hospitalized for Heart Failure

	Full Sample^a^ (*n* = 9,261)		Chart-Reviewed (*n* = 1,500)	
	Readmitted, No. (%)		Readmitted, No. (%)	
	Yes^b^ (*n* = 1,944)	No (*n* = 7,317)	Yes (*n* = 312)	No (*n* = 1,188)
Age, mean (SD), years	77.7 (9.0)	76.9 (8.5)	77.9 (8.3)	77.8 (8.7)
**Social Risk Factors**				
Lives Alone^c^	--	--	57 (18)	226 (19)
Lacks Social Support^c^	--	--	6 (2)	6 (0.5)
Marginal Housing^c^	--	--	2 (<1)	21 (2)
Substance Abuse	272 (14)	1,097 (15)	43 (14)	189 (16)
Low Income	913 (48)	3,512 (48)	146 (47)	555 (47)
**Clinical Factors**				
CABG	1,516 (78)	5,414 (74)	247 (79)	873 (73)
Heart Failure	1,846 (95)	6,951 (95)	300 (96)	1,125 (95)
Acute Coronary Syndrome	447 (23)	1,609 (22)	73 (23)	250 (21)
Coronary Atherosclerosis	1,516 (78)	5,780 (79)	243 (78)	943 (79)
Cardiopulmonary-respiratory failure and shock	447 (23)	1,975 (27)	77 (25)	311 (26)
Valvular Heart Disease	408 (21)	2,195 (30)	102 (21)	374 (31)
Arrhythmia	1,438 (74)	5,487 (75)	240 (77)	872 (73)
Other unspecified heart disease	486 (25)	1,390 (19)	69 (23)	226 (19)
Stroke	291 (15)	658 (9)	42 (13)	119 (10)
Renal Failure	1,380 (71)	4,317(59)	217 (70)	712 (60)
COPD	1,049 (54)	4,024 (55)	175 (56)	626 (53)
Diabetes	1,185 (61)	4,902 (67)	193 (62)	768 (65)
Protein-calorie malnutrition	136 (7)	292 (4)	17 (5)	41 (3)
Dementia	370 (19)	951 (13)	52 (17)	178 (15)
Functional Disability	291 (15)	878 (12)	41 (13)	131 (11)
Metastatic cancer	39 (2)	219 (3)	5 (2)	22 (2)
Major psychiatric disorders	291 (15)	878 (12)	52 (17)	135 (11)
Chronic liver disease	252 (13)	732 (10)	36 (12)	114 (10)
Severe hematologic disorders	78 (4)	220 (3)	8 (3)	30 (3)
Iron deficiency	1,069 (55)	4,024 (55)	175 (56)	649 (55)
Depression	447 (23)	1,609 (22)	79 (25)	247 (21)
Fibrosis of lung or other chronic lung disorder	136 (7)	512 (7)	19 (6)	76 (6)
Asthma	39 (2)	219 (3)	8 (3)	52 (4)
End-stage renal disease	97 (5)	220 (3)	12 (4)	18 (2)
Nephritis	136 (7)	289 (4)	25 (8)	63 (5)
Urinary tract disorders	524 (27)	1,756 (24)	86 (28)	291 (24)
Fluid and electrolyte disorders	1,049 (54)	3,365 (46)	173 (55)	538 (45)
Other psychiatric disorders	388 (20)	1,169 (16)	65 (21)	186 (16)
Peptic Ulcer and other specified GI tract disorders	369 (19)	1,243 (17)	55 (18)	173 (15)
Other GI tract disorders	1,283 (66)	4,317 (59)	201 (64)	718 (60)
Decubitus skin ulcer	213 (11)	1,024 (14)	39 (13)	168 (14)

Notes:

Abbreviations: Substance Abuse includes both drug and alcohol abuse; *CABG* coronary artery bypass graft, *COPD* chronic obstructive pulmonary disease, *GI* gastrointestinal, *MI* myocardial infarction, *PCI* percutaneous coronary intervention, *VA* Veterans Affairs; In adjusted analysis, each condition was adjusted for all respective comorbidities

^a^Full sample is the total (10,761) minus the chart-reviewed sample (1,500) with a total *n* = 9,261

^b^*p*>0.05 for all comparisons of full sample and chart-reviewed sample

^c^Variables extracted through manual chart review

Following adjustment for age (Model 1), and CMS-based predictors of readmission (Model 2), lacking social support was the only social risk factor associated with increased readmissions (Model 1: OR 5.46, 95 % CI 1.58–19.99; Model 2: OR: 4.65, 95 % CI 1.31–17.38). Marginal housing was noted to have a protective affect against readmissions in both models (Model 1: OR 0.25, 95 % CI 0.04–0.95; Model 2: OR 0.21, 95 % CI 0.03–0.87). Among clinical factors, only renal failure was associated with readmission (OR: 1.38, 95 % CI 1.02–1.87) (Table 2).

**Table 2:** Patient-level Adjusted Odds Ratios for Patients Hospitalized for Heart Failure

	Model 1: age + SRF	Model 2: CMS model + SRF
Age, mean (SD), years	1 (0.99, 1.02)	1.0 (0.98, 1.02)
Lives Alone^a^	0.92 (0.66, 1.26)	0.9 (0.64, 1.26)
Lacks Social Support^a^	5.46 (1.58, 19.99)	4.65 (1.31, 17.38)
Marginal Housing^a^	0.25 (0.04, 0.95)	0.21 (0.03, 0.87)
Substance Abuse	0.98 (0.73, 1.31)	0.91 (0.67, 1.22)
Low Income	1 (0.78, 1.29)	1.01 (0.77, 1.31)
CABG	--	1.24 (0.89, 1.74)
Heart Failure	--	1.22 (0.65, 2.47)
Acute Coronary Syndrome	--	1.06 (0.76, 1.47)
Coronary Atherosclerosis	--	0.81 (0.58, 1.14)
Cardiopulmonary-respiratory failure and shock	--	0.76 (0.55, 1.04)
Valvular Heart Disease	--	0.99 (0.74, 1.31)
Arrhythmia	--	1.1 (0.81, 1.52)
Other unspecified heart disease	--	1.09 (0.78, 1.51)
Stroke	--	1.21 (0.79, 1.84)
Renal Failure	--	1.38 (1.02, 1.87)
COPD	--	1.13 (0.86, 1.49)
Diabetes	--	0.79 (0.6, 1.05)
Protein-calorie malnutrition	--	1.41 (0.74, 2.62)
Dementia	--	0.95 (0.65, 1.38)
Functional Disability	--	1.05 (0.68, 1.61)
Metastatic cancer	--	0.73 (0.23, 1.9)
Major psychiatric disorders	--	1.39 (0.94, 2.03)
Chronic liver disease	--	1.04 (0.67, 1.59)
Severe hematologic disorders	--	0.87 (0.35, 1.94)
Iron deficiency	--	0.84 (0.63, 1.12)
Depression	--	1.08 (0.78, 1.5)
Fibrosis of lung or other chronic lung disorder	--	0.85 (0.48, 1.44)
Asthma	--	0.49 (0.21, 1.01)
End-stage renal disease	--	1.82 (0.8, 4.01)
Nephritis	--	1.42 (0.82, 2.4)
Urinary tract disorders	--	1.02 (0.75, 1.39)
Fluid and electrolyte disorders	--	1.29 (0.97, 1.71)
Other psychiatric disorders	--	1.27 (0.91, 1.77)
Peptic Ulcer and other specified GI tract disorders	--	1.16 (0.8, 1.67)
Other GI tract disorders	--	1.03 (0.77, 1.36)
Decubitus skin ulcer	--	0.68 (0.44, 1.01)

Notes

Abbreviations: Substance Abuse includes both drug and alcohol abuse; *CABG* coronary artery bypass graft, *COPD* chronic obstructive pulmonary disease, *GI* gastrointestinal, *MI* myocardial infarction, *PCI* percutaneous coronary intervention, *SRF* Social Risk Factors, *VA* Veterans Affairs; In adjusted analysis, each condition was adjusted for all respective comorbidities

^a^Variables extracted through manual chart review

In assessing model performance, a model that included age and social risks (Model 1) had a c-statistic of 0.52. A CMS-based model (age and comorbid conditions only) had a c-statistic of 0.62 - which is similar to the c-statistic of the CMS model for HF reported in the literature [11]. Adding the five social risk factors to a model that included all clinical factors in the CMS-based readmission model (Model 2) led to a c-statistic of 0.62. Model fit, as assessed by the likelihood ratio test between Model 1 and Model 2, was improved with the addition of clinical risk factors (*p*-value = 0.04). Using the HL GOF test, both models displayed no evidence of lack-of-fit between observed and expected outcomes (HL GOF: Model 1 *p*-value: 0.8, Model 2 *p*-value: 0.7).

## Discussion

Among five assessed social risk factors in a cohort of elderly Veterans hospitalized for heart failure, lacking social support was found to be associated with increased 30-day all-cause readmission while marginal housing appears to protect against readmission. Further, the inclusion of the assessed five social risk factors in a CMS-based readmission model appeared to have limited effect on model fit and performance.

Our analysis revealed several findings. First, while the estimated prevalence of a lack of social support was uncommon, it remained a moderate predictor of readmission. We hypothesize this may be due, in part, to the chart abstraction methodology we utilized. As the instances noted through chart abstraction were likely strong signals of this characteristic, hence its significant association with the outcome. Moreover, this may imply that the finding of an association with our outcomes is a conservative estimate, considering the likelihood that the lack of social support experienced among the older veterans in this study is probably significantly higher than what we described (0.5–2 %). Mechanistically, a lack of social support leading to increased readmission has face validity, as well – as those who have fewer support mechanisms may depend more on health care systems than those who have personal social support systems in place. Second, while also uncommon, marginal housing appeared to protect against readmission. We hypothesize that this may be due to the older (≥ 65 years), Veteran population that was assessed and the VA health care systems’ larger commitment to both medical and social needs of homeless Veterans [12, 13]. Thus, VA providers are more likely to discharge patients to post-acute care facilities, rather than to their own accord – limiting the patients’ probability of being readmitted in the 30-day time frame. This same mechanism may not be at play in individuals who lack social support as this quality is often difficult to ascertain and may not be shared with a provider, whereas lack of housing is a more obvious characteristic.

There are several reasons why, in this study, some social risks (lives alone, substance abuse, and low income) may not be as impactful as they are in other non-VA populations. First, the VA provides a number of social services that are commonly not offered in other health care systems, such as: subsidized transportation to/from clinics and Patient-Aligned Care Teams that focus on socially vulnerable Veterans, among many others [14, 15]. Additionally, VA patients routinely receive post-hospitalization phone calls and in-home support services, if eligible. Thus, these integrated social services may be diminishing any effect these social factors impart on readmissions risks. Moreover, the only risk factor we found associated with increased 30-day readmission was lack of social support – which is a difficult risk factor for a health system to intervene upon. Second, while we assessed a variety of common risk factors shown to be influential in other settings, these specific factors may not play a tangible role in patients with heart failure – as prior studies have shown social risk variables are not universally impactful across all disease states [16]. Assessing other risks, such as access to transportation and medical literacy, which are also suspected to impact HF readmissions [17], would be beneficial. Third, the fidelity of the social risk variables could be playing a role in the low prevalence – as previous work has shown that social risk factors are substantially more prevalent than represented in administrative data [18]. We attempted to address this issue by extracting three risk factors through manual chart review, noting that the only factors shown to be associated with readmission (lack of social support and marginal housing) were those extracted in this method. Finally, the limited sample size may be impacting our ability to capture a signal with some risk factors. Though the sample size is modest, our approach did allow for manual chart abstraction of three social risk factors.

This study does have methodologic limitations. First, the cohort come from a 2012 data set which allowed us the ability to perform the chart review portion of this assessment. While this may impact the current validity of our findings, we note that studies have shown that underlying social determinants have remained impactful and present over long periods of time in the US [19]. Second, chart abstraction may not be sensitive enough to detect the presence of the assessed social risk factors – as this information may not be viewed as relevant to patient care, and thus, not documented in the chart by the provider. Third, there may be non-VA based social support mechanisms in place (e.g., meals-on-wheels) that could be impacting our findings that we were unable to ascertain or control for. Finally, the observational nature of this work does not allow us to speculate beyond associations, and the focus on elderly Veterans, limits generalizability.

## Conclusions

In an elderly population of Veterans, lacking social support may be associated with increased risk of 30-day readmission in patients hospitalized for HF. Additionally, the inclusion of the five measured social risk factors (living alone, lack of social support, marginal housing, substance abuse, and low income) into a CMS-based readmission model does not improve model performance. The VAs integrated health care system, where many social risk factors are addressed through national programs, may be attenuating the deleterious impact some social risk factors have on hospital readmission.

## Supplementary Information

**Additional file 1.**

## Data Availability

The data that support the findings of this study are available from the Veterans Health Administration, but restrictions apply to the availability of these data, which were used under license for the current study, and so are not publicly available.

## Abbreviations

CMS: Centers for Medicare & Medicaid
HF: Heart Failure
HL GOF: Hosmer-Lemeshow goodness-of-fit
ICD-9-CM: International Classification of Disease, Ninth Revision, Clinical Modification
MedPAR: Medicare Provider Analysis and Review
PACT: Patient-Aligned Care Teams
VA: Veterans Health Administration
VIReC: VA Information Resource Center
