# Supplementary material for: Examining the association of social risk with heart failure readmission in the Veterans Health Administration
Source: BMC Health Serv Res. 2021 Aug 26;21:874. doi: 10.1186/s12913-021-06888-1 (PMC8393433; doi:10.1186/s12913-021-06888-1)
Supplement: Supplementary file 1 — Additional file 1. [file 12913_2021_6888_MOESM1_ESM.docx]

**SUPPLEMENTAL MATERIAL**

**1) HOUSING_SITUATION**

We define a patient-level classification for **HOUSING_SITUATION** as any information representing homelessness or the patient’s current housing situation.

We define homelessness as follows:

1. An individual who lacks a fixed, regular, and adequate nighttime residence;
2. An individual who has a primary nighttime residence that is:
   1. a supervised publicly or privately operated shelter designed to provide temporary living accommodations (this includes welfare hotels, congregate shelters, and transitional housing for the mentally ill);
   2. a public or private place not designed for, or ordinarily used as, a regular sleeping accommodation for human beings.

Any mentions of confirmed homelessness where the veteran is currently experiencing homelessness should be considered. We are interested in the **current housing situation**, not a history of prior homelessness. If the veteran has a “Form X” as part of their record (a template questionnaire), this is considered information that represents confirmed homelessness (if current).

Each classification for **HOUSING_SITUATION** should be defined using the following criteria:

**Questions to ask:**

1. **Is there direct evidence that the patient is not homeless or living at home**

**e.g. (lives at home with wife, lives with daughter)**

1. **Is there a direct mention of homelessness?**

Ex. Direct mentions of “**homeless”**, **“homelessness”**, etc.

Ex. “This 40-year-old, **[homeless],** white male was admitted to the ER yesterday evening.”

Ex: “Other issues: **[homelessness],** unemployed.”

Ex. Living in a shelter

1. **Is the patient at risk of becoming homeless/or marginally housed?**

Ex. “patient is **[struggling to pay rent]**”

Ex. “He is currently **[sleeping on his girlfriend’s couch]**”

Ex. “He has been **[living with his girlfriend]** over the last month. Ex. Not yet homeless but can’t pay rent

1. **Is the patient living in some type of care facility?**

Ex. “patient currently resides in a **[CLC]”… [nursing home], [assisted care facility] [group home]**

1. **Is the patient living in permanent single room occupancy (SRO)?**
2. **No mention of the patients housing situation.** This is the default value.

**Exclusion criteria:**

- **Exclude information that is not asserted or not associated with the patient.**

Ex. “Homelessness: N/A”

Ex. “Patient stated that a few of his friends committed suicide or became homeless”.

Ex. “She said she was doing fine and there was no need to talk to a homeless coordinator at this point”.

**2) LIVING_ALONE**

We define a patient-level classification for **LIVING_ALONE** as any information indicating that the patient lives by themselves without the presence of others who cohabitate in the same space. The **most recent** living situation relative to the **index hospital admission** should be identified.

Each classification for **LIVING_ALONE** should be defined using the following criteria:

**Questions to ask:**

1. **Is there evidence that the patient lives alone?**

Ex. “Patient **[lives alone]**”

1. **Is there evidence that the patient does not live alone?**

Ex. “patient **[lives with friends/relatives]”**

Ex. “patient **[lives with wife]”**

Ex. “patient **[lives with son]”**

1. **Is there evidence that the patient lives in a group setting such as a nursing home, senior care center, nursing home, community living center?**

Ex. “Patient currently [**resides in a nursing home]**”

1. **No mention of the patients living situation.** This is the default value.

**Exclusion criteria:**

- **Exclude information that is not asserted or not associated with the patient.**

**3) SOCIAL_SUPPORT**

We define a patient-level classification for **SOCIAL_SUPPORT** as any information that indicates a support mechanism or social support status including mentions of wife or husband being involved, children being involved, neighbors or friends being involved, church groups involvement or other organizations that provide social support mechanisms.

Evidence of social support or lack of social support includes:

1. Evidence of social support includes mentions that someone is involved in the care of the patient: If there is a mention that the patient has a supportive and involved family, neighbors or friends then the patient has evidence of social support. ***Just stating a patient is married or has children is not evidence of social support*;** there must be evidence that these family members are in some way engaged with patient (e.g., call the patient), call the patients doctor, or involved in care (e.g., manage medications, feed the patient, bring them to appointments, pick them up from the hospital, make food, help with cleaning etc..)
2. Evidence of involvement and engagement in a patient’s life. We will take mentions of involvement in someone’s life (e.g., “meets up with friends”, “has a supportive family”, “children see him often”, “very involved with church group”) as evidence of social support. For example, a relatively healthy patient may not need someone to drive them to the doctor and can manage their own meds but may have lots of social support as evidenced by lots of people in their life. ***Again I would not take a mention of having children or being married as social support because many patients have children and partners that are not engaged in their life.***
3. Evidence of lack of social support: *Direct evidence* includes mentions of social isolation in the chart or explicit mentions of no family or friends involved in life. If a provider explicitly states the patient is “socially isolated” then we will take that as “lacks social support”. *Indirect evidence of lack of social support* could be that no one is available to pick up the patient from the hospital or bring them to the clinic or be available to pick up after a procedure that involves sedation.

Temporary post hospitalization services such as a nurse or physical therapist that comes in post hospitalization for only 2 weeks is ***NOT*** evidence of social support.

****If the only mention of having support is “lives with wife” or “lives with son”, that should be coded in LIVING SITUATION and the social support should be coded as “no mention”**

Each classification for **SOCIAL_SUPPORT** should be defined using the following criteria:

**Questions to ask:**

1. **Does the patient have some form of social support?**

Ex. “[Relation/friend brings patient to clinic]”

Ex. “[Relation/friend manages medications]”

Ex. “[Neighbor checks in on him and brings food]”

Ex. “[Relation/friend manages helps with laundry and cleaning]”

Ex. “[Has lots of family and friends who keep in touch with him]”

Ex. “[meets up with friends regularly]”

Ex. “[very close to family who visit often]”

Ex. “[has 3 kids, two who live close by and see him regularly”

Ex. “[Friend brings him to the doctor]”

Ex. “[Has a permanent caregiver] that comes 2 times a week”

1. **Is there evidence that the patient lacks social support?**

Ex. “[Family not yet engaged with care]”

Ex. “[patient is socially isolated]”

Ex. “[no one to pick the patient up on hospital discharge]”

Ex. “[has no one to bring him to the doctor]”

1. Is there evidence that the patient has access to community services:

Ex meals on wheels

Ex. Church services

1. **No mention of patient social support.** This is the default value.

**Exclusion criteria:**

- **Exclude information that is not asserted or not associated with the patient.**

1. **Attribute assignment**

In this section, we describe how to correctly encode attributes necessary for interpreting or normalizing the underlying context of a mention.

**We define the following encodings:**

1. Encoding a ***housingnorm*** attribute – patient-level classification for **Housing_Situation** will be normalized to the following mutually exclusive values:
   1. homeless
   2. marginally housed/temporarily housed/ at risk of homelessness
   3. lives at home/not homeless (not homeless but other housing situation, for example “lives with son”)
   4. lives in a permanent single room occupancy
   5. living in a nursing home
   6. living in an assisted living facility
   7. living in group home
   8. no mention (default)
2. Encoding a ***livingalonenorm*** attribute – patient-level classification for **Living_Alone** will be normalized to the following mutually exclusive values:
   1. living alone
   2. does not live alone
   3. lives in a group setting
   4. no mention (default)
3. Encoding a **socialsupportnorm** attribute – patient-level classification for **Social_Support** will be normalized to the following mutually exclusive values:
   1. has social support
   2. no social support
   3. has access to community services
   4. no mention (default)
